# Accuracy of intraoperative aberrometry versus preoperative biometry for intraocular lens power selection in short and long eyes

**DOI:** 10.3389/fmed.2024.1466885

**Published:** 2024-10-15

**Authors:** Pedro Tañá-Rivero, Paz Orts-Vila, Pedro Tañá-Sanz, Santiago Tañá-Sanz, Ramón Ruiz-Mesa, Robert Montés-Micó

**Affiliations:** ^1^Oftalvist, Alicante, Spain; ^2^Oftalvist, Jerez de la Frontera, Spain; ^3^Optics and Optometry and Vision Sciences Department, University of Valencia, Valencia, Spain

**Keywords:** intraoperative aberrometry, short, long, intraocular lens, phacoemulsification, cataract

## Abstract

**Background:**

To compare the accuracy of intraoperative wavefront aberrometry using the ORA VLynk system with different biometry-based formulas in short and long eyes after cataract surgery.

**Methods:**

This prospective study considered 48 eyes with axial lengths of <22.1 mm and 48 eyes with axial lengths of >25.0 mm. All eyes were implanted with the monofocal AcrySof IQ IOL, the power being determined using the ORA VLynk. The postoperative spherical equivalent (SE) at 3 months was compared to that predicted preoperatively using the SRK/T, Hoffer Q, Haigis, Holladay 2, Barrett Universal II, and Barrett True K formulas and intraoperatively using the ORA VLynk. Mean numerical and absolute errors and the percentage of eyes within ±0.50 D/1.00 D of their target were obtained.

**Results:**

For long eyes, the mean absolute error values were 0.35, 0.52, 0.34, 0.30, 0.29, 0.27, and 0.24D for SRK/T, Hoffer Q, Haigis, Holladay 2, Barrett Universal II, Barrett True K, and ORA VLynk, respectively (*p* < 0.001). These values were 0.55, 0.45, 0.49, 0.40, 0.44, 0.44 and 0.50 D for short eyes, respectively (*p* < 0.001). The proportions of long eyes within ±0.50 D of the target were 77.08, 50, 75, 85.42, 83.33, 79.17, and 87.50%, respectively; and 50, 66.67, 60.42, 66.67, 60.42, 60.42, and 58.33%, respectively, for short eyes.

**Conclusion:**

The ORA VLynk performs better than all biometry-based formulas in long eyes and, in short eyes, it is as effective as SRK/T, Haigis, Barrett Universal II, and Barrett true K, with the Hoffer Q and Holladay 2 being the most accurate; however, the differences between the calculation methods were small.

**Clinical trial registration:**

Identifier DRKS000028106.

## 1 Introduction

Cataract surgeons frequently see patients who have been submitted to previous corneal refractive surgeries such as radial keratotomy, photorefractive keratectomy (PRK) or laser *in situ* keratomileusis (LASIK). In this type of patient, the intraocular lens (IOL) power calculation is more challenging despite the use of next generation formulas and/or available calculators. The use of intraoperative wavefront aberrometry, utilised by many surgeons, may help to provide patients with the best possible refractive and visual outcomes. This has proved useful in post-PRK/LASIK and eyes with radial keratotomy (1–8). This technology has also been shown to be beneficial in non-post-refractive surgery eyes (9) and eyes with low (10) or high (11) amounts of corneal astigmatism.

The Optiwave Refractive Analysis System (ORA, Alcon Laboratories, Inc.; Fort Worth, TX, United States) is an intraoperative wavefront aberrometry system that measures the whole refractive system (anterior and posterior cornea) allowing surgeons to determine the IOL power required for the eye. In addition, ORA may be useful in other situations in which IOL power calculations are difficult, for example eyes with high axial myopia or hyperopia. Several clinical studies have published refractive outcomes using the ORA system versus preoperative biometry to select IOL power for short and long eyes (12–16). These studies compare the accuracy of the ORA system with conventional biometry-based formulas in eyes implanted with different types of IOLs: monofocal, toric, and multifocal. To our knowledge, no prospective studies have assessed the accuracy of the ORA VLynk and preoperative biometry formulas in short and long eyes when the same IOL was implanted.

The main purpose of this study was, therefore, to compare the accuracy of intraoperative aberrometry using the ORA VLynk system with different conventional biometry-based formulas in short and long eyes implanted with the same monofocal IOL after cataract surgery. The postoperative refraction was compared with the preoperative and intraoperative predictions in order to evaluate the accuracy of each method.

## 2 Materials and methods

This prospective comparative clinical study was approved by the Ethics Committee at Investigación con Medicamentos de Cádiz (Cádiz, Spain) and the Valencia regional committee on postmarketing studies, CAEPRO (Valencia, Spain). All the procedures adhered to the tenets of the Declaration of Helsinki, patients recruited to the study provided written informed consent before they were enrolled, and the study was registered in the German Clinical Trials Register (DRKS000028106). The inclusion criteria were patients over 40 years of age who were willing and able to attend the study visits, who presented cataract or refractive lens exchange with an axial length of either <22.1 mm or > 25.0 mm and valid ORA VLynk measurements taken during the surgery.

The exclusion criteria were corneal opacity, previous radial keratotomy or other corneal surgery, previous anterior or posterior chamber surgery, vitrectomy, laser iridotomy, diabetic retinopathy, history of retinal detachment, patients with acute or chronic disease, keratoconus, amblyopia and/or strabismus, and pregnancy. All patients included in the study underwent a complete ophthalmological examination with routine cataract evaluation measurements measuring Snellen decimal monocular best-corrected distance visual acuity (CDVA), manifest refraction, and optical biometry performed with the IOLMaster 700 swept source optical coherence tomographer (Carl Zeiss Meditec AG, Jena, Germany). The IOL power calculation was based on this measurement considering the SRK/T, Hoffer Q, Haigis, Holladay 2, Barrett Universal II, and Barret True K formulas for all eyes. The predicted postoperative spherical equivalent (SE) was calculated for each condition. In addition, all patients underwent ORA VLynk analysis, which also generated a predicted postoperative SE that was used for comparison. The power of the implanted IOL was determined using the ORA VLynk. The targeted refraction in all cases was emmetropia.

Phacoemulsification was performed using the Centurion Vision System (Alcon Laboratories, Inc.; Fort Worth, TX, United States) through a 2.2-mm temporally located clear corneal incision considering a historical level of surgically induced astigmatism by an incision of <0.25 D. A 5 mm diameter circular anterior capsulotomy centred on the capsular bag was performed and, after cataract removal and posterior capsule polishing, the capsular bag was filled with 1.0% sodium hyaluronate (Provisc, Alcon Laboratories, Inc.; Fort Worth, TX, United States). The AcrySof IQ monofocal IOL (Alcon Laboratories, Inc.; Fort Worth, TX, USA) was implanted in all the eyes. The postoperative examination at 3 months post-surgery included CDVA and manifest refraction measurements.

The primary outcome measurements included the difference between the predicted target and the actual postoperative SE for each method. This difference is referred to as the mean arithmetic error. The mean absolute error (absolute value of the arithmetic error) and median absolute error were also calculated. The secondary endpoint included the proportion of eyes within ±0.25 D, ±0.50 D, ±0.75 D and ± 1.00 D of the SE target refraction for each method.

### 2.1 Statistical analysis and sample size

The statistical analysis was carried out using SPSS software (22.0 version, IBM Corp., Armonk, New York, United States). All the measurements are shown as the mean ± standard deviation (SD). The normality of the distribution was checked using the Shapiro–Wilk test. Statistically significant differences between the different calculation methods were assessed using Friedman repeated measures analysis of variance. The Tukey test was used for post-hoc analysis to compare the data between methods whenever the Friedman test revealed significant differences between the values obtained. This test gave us the significance level for paired differences between the individual conditions of comparison between methods. The statistical significance limit was set to a *p* value of <0.05 in all cases. Data from a similar study (14) was used to compute the required sample size for an analysis of variance model with 1 group, 5 repetitions, a statistical power of 0.9, a significance of 0.05 and an estimated correlation among repeated observations of 0.8. Given these conditions, the minimum required sample size was 23 independent observations for each group; for this reason, a target cohort of 25 subjects per group was considered large enough to account for potential dropouts.

## 3 Results

In this study all eyes (*n* = 96) were implanted with the same IOL, the AcrySof IQ IOL. Table 1 shows the main characteristics of the study population. 53 patients (34 females) with a mean age of 71.94 ± 8.18 years were included in the study. There were no complications in any of the cases during surgery and follow-up.

**Table 1:** Demographic characteristics and preoperative measurements of participants shown as means, standard deviations (SD) and ranges.

	Long eyes	Short eyes
Eyes (*n*)	48	48
Sphere (D)	−5.81 ± 4.59 (−16.50 to 0.50)	2.28 ± 2.61 (−3.00 to 8.00)
Refractive Cylinder (D)	−1.32 ± 0.80 (0 to −3.00)	−0.84 ± 0.74 (0 to −2.75)
Spherical Equivalent (D)	−6.47 ± 4.57 (−16.50 to 0.25)	1.86 ± 2.47 (−3.00 to 7.13)
CDVA (decimal)	0.62 ± 0.26 (0.10 to 1.00)	0.59 ± 0.25 (0.05 to 1.00)
IOP (mmHg)	13.19 ± 3.58 (6.00 to 17.50)	17.61 ± 4.70 (11.00 to 32.00)
K1 (D)	43.16 ± 1.14 (40.67 to 45.67)	44.74 ± 1.73 (40.58 to 48.92)
K2 (D)	44.17 ± 1.17 (41.62 to 46.88)	45.73 ± 1.63 (42.76 to 49.54)
Axial length (mm)	26.23 ± 1.18 (25.01 to 29.24)	21.65 ± 0.45 (20.03 to 22.05)
ACD (mm)	3.55 ± 0.48 (2.35 to 5.55)	2.69 ± 0.35 (2.01 to 3.23)
LT (mm)	4.53 ± 0.39 (3.87 to 5.61)	4.81 ± 0.46 (2.74 to 5.33)
WTW (mm)	12.11 ± 0.36 (11.40 to 12.90)	11.59 ± 0.33 (10.90 to 12.20)
IOL power (D)	13.19 ± 3.58 (6.00 to 17.50)	26.18 ± 1.94 (22.00 to 30.00)

CDVA: corrected distance visual acuity; IOP: intraocular pressure; K: keratometry; ACD: anterior chamber depth; LT: lens thickness; WTW: white-to-white; IOL: intraocular lens power.

**Figure 1:**
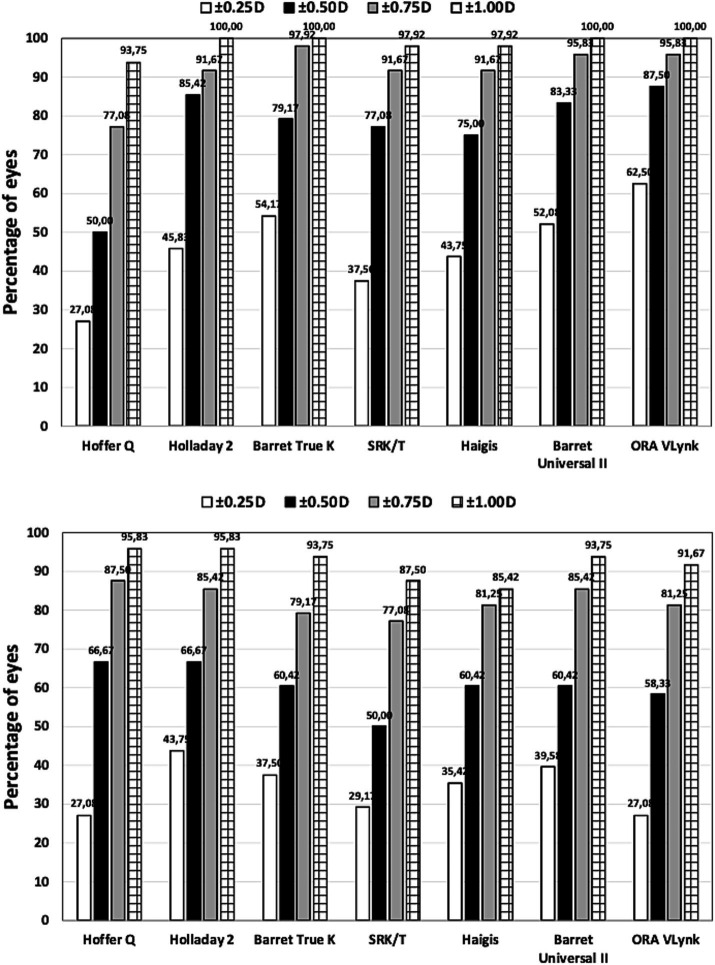
Proportion of eyes within ±0.25 D, ±0.50 D, ±0.750 D and ± 1.00 D of the target spherical equivalent refraction for long (top) and short (bottom) eyes using different calculation methods.

**Figure 2:**
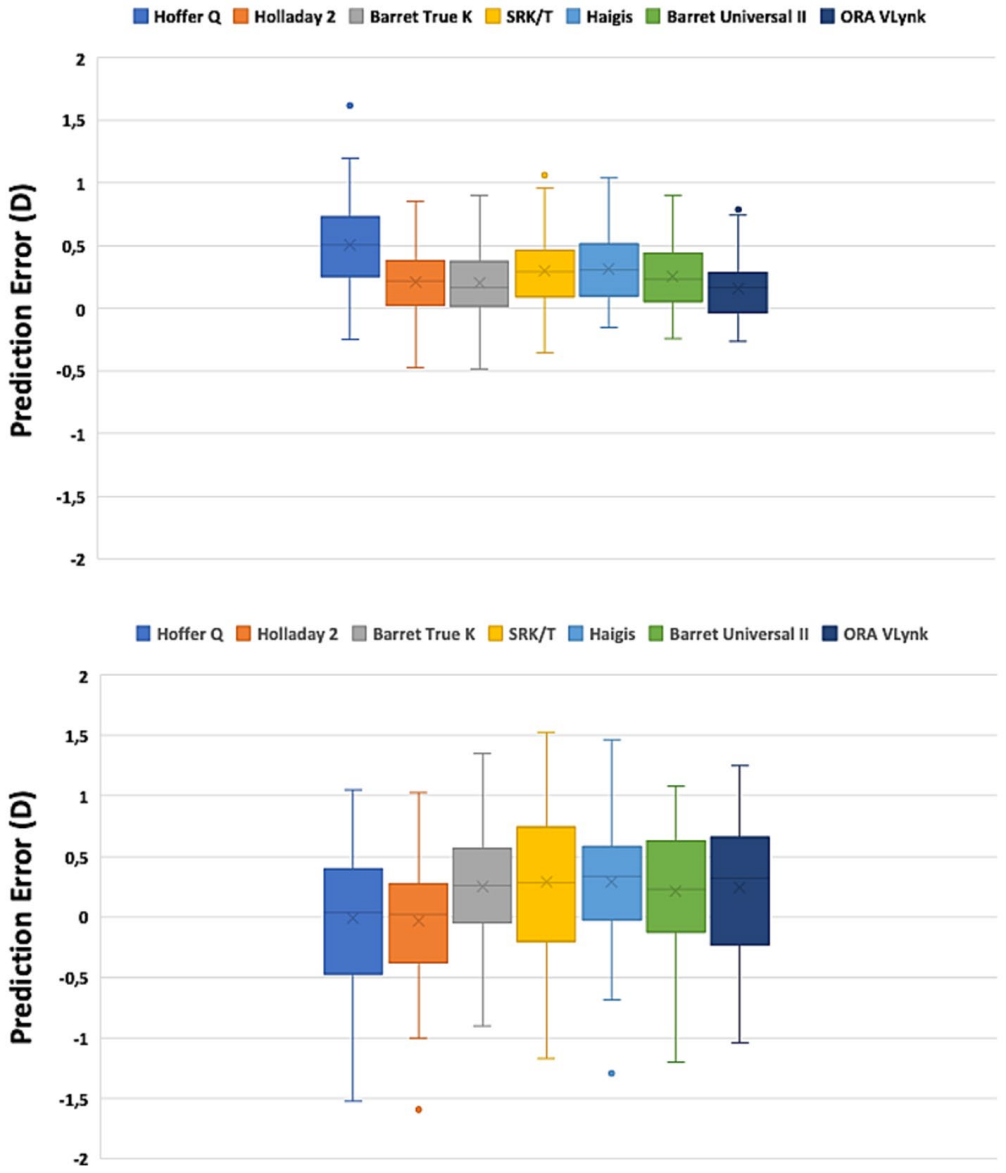
Box plot graph for the long (top) and short (bottom) eyes using different calculation methods.

The mean residual SE was −0.05 ± 0.31 D for long eyes and 0.10 ± 0.53 D for short eyes. The preoperative and postoperative CDVAs for long eyes were 0.62 ± 0.26 and 0.96 ± 0.12, respectively, and 0.59 ± 0.26 and 0.94 ± 0.16, for short eyes. There was statistically significant postoperative improvement in CDVA (*p* < 0.001). Table 2 was created to compare the accuracy between the ORA VLynk and the IOL calculation formulas. This table shows the outcomes reported for the different methods using the mean error, mean absolute error and median absolute error. Figure 1 shows the proportion of eyes within ±0.25 D, ±0.50 D, ±0.750 D and ± 1.00 D of the target SE refraction and Figure 2 the interquartile range. It indicates that for long eyes the ORA VLynk performs better than all the other IOL calculation formulas with the minimum value for the mean absolute error (0.24 D) and median absolute error (0.18 D,) and the highest percentages of eyes within ±0.50 D (87.50%) and ± 1.00 D (100%). For short eyes, the Holladay 2 IOL formula performed best, with a mean absolute error of 0.40 D, a median absolute error 0.28D, and 66.67 and 95.83% of eyes for ±0.50 D and ± 1.00 D, respectively.

**Table 2:** Outcomes (mean ± standard deviation and range) reported using the different calculation method for long and short eyes.

Method	Mean error	Mean absolute error	Median absolute error
Long eyes			
SRK/T	0.29 ± 0.30 (−0.35 to 1.06)	0.35 ± 0.24 (0.02 to 1.06)	0.31
Hoffer Q	0.51 ± 0.36 (−0.25 to 1.62)	0.52 ± 0.33 (0.02 to 0.86)	0.50
Haigis	0.31 ± 0.28 (−0.15 to 1.04)	0.34 ± 0.25 (0.01 to 1.04)	0.30
Holladay 2	0.21 ± 0.31 (−0.47 to 0.86)	0.30 ± 0.22 (0.02 to 0.86)	0.28
Barrett Universal II	0.26 ± 0.26 (−0.24 to 0.90)	0.29 ± 0.23 (0.02 to 0.90)	0.24
Barrett true K	0.21 ± 0.28 (−0.49 to 0.90)	0.27 ± 0.22 (0.01 to 0.90)	0.22
ORA VLynk	0.16 ± 0.27 (−0.27 to 0.83)	0.24 ± 0.20 (0.01 to 0.83)	0.18
*P* value	<0.001	<0.001	
Short eyes			
SRK/T	0.29 ± 0.60 (−1.17 to 1.53)	0.55 ± 0.37 (0.01 to 1.53)	0.52
Hoffer Q	−0.01 ± 0.54 (−1.53 to 1.05)	0.45 ± 0.30 (0.01 to 1.52)	0.40
Haigis	0.29 ± 0.54 (−1.29 to 1.47)	0.49 ± 0.37 (0.01 to 1.47)	0.45
Holladay 2	−0.03 ± 0.52 (−1.59 to 1.03)	0.40 ± 0.33 (0.00 to 1.59)	0.28
Barrett Universal II	0.21 ± 0.51 (−1.20 to 1.08)	0.44 ± 0.33 (0.01 to 1.20)	0.44
Barrett true K	0.25 ± 0.50 (−0.90 to 1.35)	0.44 ± 0.33 (0.00 to 1.35)	0.36
ORA VLynk	0.24 ± 0.54 (−1.04 to 1.25)	0.50 ± 0.31 (0.00 to 1.25)	0.46
*P* value	<0.001	<0.001	

Since we found a statistically significant difference between the different mean errors, a Tukey test for pairwise analysis was run on this parameter to discover the differences between the calculation methods. The outcomes obtained for long and short eyes are shown in Table 3. For long eyes, the ORA VLynk had the lowest mean numerical error and the difference was statistically significant when compared to the biometry-based formulas, except for Holladay 2 (*p* = 0.272). Hoffer Q performed statistically worse than the other biometry-based formulas and ORA VLynk (*p* ≤ 0.007). For short eyes, the Hoffer Q and Holladay 2 formulas had the lowest mean numerical error and were not significantly different from one another (*p* = 0.932). Specifically, the outcomes of the ORA VLynk were comparable with those of SRK/T, Haigis, Barrett Universal II, and Barrett true K (*p* > 0.9).

**Table 3:** Post hoc analysis using the different calculation method for long and short eyes.

	*P* value						
Method	SRK/T	Hoffer Q	Haigis	Holladay 2	Barrett Universal II	Barrett True K	ORA VLynk
Long eyes							
SRK/T	—	—	—	—	—	—	—
Hoffer Q	<0.001*	—	—	—	—	—	—
Haigis	0.961	0.007*	—	—	—	—	—
Holladay 2	0.260	<0.001*	0.021*	—	—	—	—
Barret Universal II	0.996	<0.001*	0.694	0.647	—	—	—
Barrett true K	0.939	<0.001*	0.395	0.893	0.999	—	—
ORA VLynk	<0.001*	<0.001*	<0.001*	0.272	0.002*	0.010*	—
Short eyes							
SRKT/	—	—	—	—	—	—	—
Hoffer Q	<0.001*	—	—	—	—	—	—
Haigis	1.000	<0.001*	—	—	—	—	—
Holladay 2	<0.001*	0.932	<0.001*	—	—	—	—
Barrett Universal II	0.973	<0.001*	0.961	<0.001*	—	—	—
Barrett true K	1.000	<0.001*	1.000	<0.001*	0.995	—	—
ORA VLynk	0.999	<0.001*	0.998	<0.001*	1.000	1.000	—

*Statistically significant.

## 4 Discussion

Previously published clinical studies have pointed out the benefit of using intraoperative wavefront aberrometry in long and short eyes. Table 4 shows the main characteristics of studies that used the ORA system, indicating the axial length considered, the number of eyes included, the formulas used, the type of IOLs implanted, and postoperative follow-up. All of these, except for Bansal et al. (16) and our study, were retrospective.

**Table 4:** Clinical studies using the Optiwave Refractive Analysis System (ORA) in short and long eyes.

Authors	Year	Axial length (mm)	Eyes	Formulas	IOL implanted	Follow up
Hill et al. (12)	2017	>25.0	51	SRK/T Holladay 1 A-optimized Holladay 1 Holladay 2 Barrett Universal II Hill-RBF	30 eyes with monofocal IOL (Akreos AO60, AF-1 FY-60 AD or AcrySof MN60MA) 13 eyes with toric IOL (Tecnis ZCT150, Tecnis ZCT225, Tecnis ZCT300, or Tecnis ZCT400) 8 eyes with multifocal IOL (Tecnis ZMB00)	21–60 days
Sudhakar et al. (13)	2019	<22.1	51	Hoffer Q Holladay 2 Haigis Barrett Universal II Hill-RBF	37 eyes with monofocal IOL (Akreos AO60, AF-1 FY-60 AD or SA60AT) 9 eyes with toric IOL (Tecnis ZCT150, ZCT225, ZCT300 or ZCT400) 5 eyes with multifocal IOL (Tecnis ZKB00 or ZLB00)	20–60 days
Soifer et al. (14)	2021	≥25 ≥27 <22	107 14 23	Barrett Universal II	NA	4 weeks or later
Sakai et al. (15)	2022	≥25	61 39* 22**	SRK/T Holladay 1 Hoffer Q Holladay 2 Haigis Barrett Universal II	13 eyes with monofocal IOL 20 eyes with toric IOL 28 eyes with multifocal IOL	1 week-2 months
Bansal et al. (16)	2022	<22	65	SRK/T Hoffer Q Haigis Holladay 2 Barrett Universal II Hill-RBF	All eyes with monofocal IOL (59 with AcrySof IQ and 6 with AcrySof SA60AT)	4 weeks
Current study	2023	>25.0 <22.1	48 48	SRK/T Hoffer Q Haigis Holladay 2 Barrett Universal II Barret True K	All eyes with monofocal IOL (AcrySof IQ)	3 months

IOL: intraocular lens; NA: not available; *: emmetropia target (0 to − 0.50D); **: myopia target (−2.00 to − 5.00D).

In our work, the improved performance shown by the ORA Vlynk for long eyes compared to the biometry-based formulas was expected and is consistent with the findings reported by other studies. Three studies have been carried out on long eyes (see Table 3 for details). In the first, Hill et al. (12) used 51 eyes with an axial length of >25.0 mm to retrospectively compare the accuracy of ORA with several formulas. They concluded that ORA was better than all formulas based on preoperative biometry and as effective as the AL-optimised Holladay 1 formula in predicting residual refractive error and reducing hyperopic outcomes. Specifically, they also indicated that the performance of Hill-RBF was similar to that of the fourth-generation formulas. It should be noted that they analysed the mean numerical error and not the mean absolute error when comparing accuracy. When compared to our study, only mean numerical error, the outcomes were found to be quite similar (within about a quarter of a diopter, see Table 5). We fully agree with this study since our outcomes revealed that the ORA VLynk had the lowest mean numerical error and the difference from the biometry-based formulas was statistically significant, except for Holladay 2 (*p* = 0.272). In the second study, Sakai et al. (15) also retrospectively compared this technique with IOL calculation formulas in eyes with axial lengths of ≥25 mm with emmetropic (0 to −0.50D, *n* = 39) and myopic (−2.00 to −5.00D, *n* = 22) targets. ORA was revealed to be the most accurate method for predicting postoperative refraction in eyes with an emmetropic target, whereas the Barrett Universal II formula was found to be the most accurate for eyes with a myopic target. These authors also indicated that a myopic shift in the refractive outcome should be considered when ORA is used to target myopia. Soifer et al. (14) analysed 121 highly myopic eyes to assess whether ORA improves the accuracy compared to the Barrett Universal II formula. They concluded that ORA demonstrated similar refractive results to the Barrett Universal II formula, and may provide an additional benefit for eyes with an axial length of ≥27 mm. Our results, comparing the mean absolute error, were better than those found by these authors (see Table 5), with the ORA VLynk being significantly more accurate than the Barrett Universal formula II (see Table 3; *p* = 0.002). In eyes with a long axial length, hyperopic surprise has often been reported. Yokoi et al. (17) evaluated the refractive error after cataract surgery in 568 highly myopic eyes (≥26.50 mm) selecting the IOL power with the SRK/T formula and reported a mean refractive error of +0.45 ± 0.79 D and a mean absolute refractive error of +0.72 ± 0.47 D, with 70% of the refractive errors being within ±1.00 D of the targeted refraction. Their findings showed that the postoperative refractive error was significantly greater in eyes whose axial length was ≥31.0 mm than in eyes with shorter axial lengths. The outcomes of our study show small postoperative mean errors.

**Table 5:** Mean numerical error (mean absolute error) reported in different clinical studies using several calculation methods.

Authors	SRK/T	Holladay 1	A-optimized Holladay 1	Holladay 2	Barrett Universal II	Hill-RBF	Hoffer Q	Haigis	Barret true K	ORA VLynk
*Long eyes*										
Hill et al. (12)	0.20 ± 0.06 (NA)	0.33 ± 0.06 (NA)	−0.02 ± 0.06 (NA)	0.24 ± 0.06 (NA)	0.19 ± 0.06 (NA)	0.22 ± 0.06 (NA)				0.06 ± 0.06 (NA)
Sakai et al. (15)*	NA (0.38 ± 0.36)	NA (0.59 ± 0.40)		NA (0.47 ± 0.37)	NA (0.35 ± 0.33)		NA (0.56 ± 0.39)	NA (0.44 ± 0.35)		0.04 ± 0.39 (0.28 ± 0.27)
Current study	0.29 ± 0.30 (0.35 ± 0.24)			0.21 ± 0.31 (0.30 ± 0.22)	0.26 ± 0.26 (0.29 ± 0.23)		0.51 ± 0.36 (0.52 ± 0.33)	0.31 ± 0.28 (0.34 ± 0.25)	0.21 ± 0.28 (0.27 ± 0.22)	0.16 ± 0.27 (0.24 ± 0.20)
Short eyes										
Sudhakar et al. (13)				−0.14 ± NA (0.53 ± NA)	0.11 ± NA (0.51 ± NA)	0.07 ± NA (0.49 ± NA)	−0.08 ± NA (0.54 ± NA)	0.26 ± NA (0.60 ± NA)		0.00 ± NA (0.48 ± NA)
Bansal et al. (16)†	−0.02 ± 0.56 (0.46 ± 0.32)			−0.23 ± 0.66 (0.54 ± 0.44)	0.01 ± 0.60 (0.49 ± 0.34)	−0.06 ± 0.53 (0.40 ± 0.35)	−0.24 ± 0.55 (0.42 ± 0.42)	−0.20 ± 0.82 (0.63 ± 0.56)		−0.10 ± 0.50 (0.37 ± 0.35)
Current study	0.29 ± 0.60 (0.55 ± 0.37)			−0.03 ± 0.52 (0.40 ± 0.33)	0.21 ± 0.51 (0.44 ± 0.33)		−0.01 ± 0.54 (0.45 ± 0.30)	0.29 ± 0.54 (0.49 ± 0.37)	0.25 ± 0.50 (0.44 ± 0.33)	0.24 ± 0.54 (0.50 ± 0.31)

*Target emmetropia; †without optimization.

Table 6 shows the proportion of eyes within ±0.50 D and ± 1.00 D of the target spherical equivalent refraction reported in different clinical studies using several calculation methods. For long eyes, our results showed slightly higher percentages compared to those found by Hill et al. (12), Soifer et al. (14), and Sakai et al. (15). We found the best outcomes for the ORA Vlynk, in agreement with the findings of Soifer et al. (14). Hill et al. (12) found best percentage outcomes for the A-optimised Holladay 1 formula (82.4 and 100% for ±0.50 D and ± 1.00 D, respectively).

**Table 6:** Proportion of eyes within ±0.50D (±1.00D) of the spherical equivalent target refraction reported in different clinical studies using several calculation methods.

Authors	SRK/T	Holladay 1	A-optimized Holladay 1	Holladay 2	Barrett Universal II	Hill-RBF	Hoffer Q	Haigis	Barret true K	ORA VLynk
Long eyes										
Hill et al. (12)	74.5 (96.1)	62.8 (90.2)	82.4 (100)	79.1 (90.7)	73.9 (96.1)	76.7 (93.0)				80.4 (98.0)
Soifer et al. (14) ≥25 mm ≥27 mm					79.4 (96.3) 64.3 (92.9)					75.7 (98.1) 71.4 (92.9)
Sakai et al. (15)										84.6 (97.4)
Current study	77.08 (97.92)			85.42 (100)	83.33 (100)		50.00 (93.75)	75.00 (97.92)	79.17 (100)	87.50 (100)
Short eyes										
Sudhakar et al. (13)				43.1 (88.2)	52.9 (86.3)	60.8 (90.2)	49.0 (86.3)	52.9 (80.4)		58.8 (88.2)
Soifer et al. (14)					52.2 (91.3)					52.2 (87.0)
Bansal et al. (16)†	63.08 (93.85)			53.85 (80.00)	60.0 (95.38)	70.77 (96.92)	69.23 (93.85)	50.77 (84.62)		67.69 (95.38)
Current study	50.00 (87.50)			66.67 (95.83)	60.42 (93.75)		64.58 (95.83)	60.42 (85.42)	60.42 (93.75)	58.33 (91.67)

†Without optimization.

Additionally, three studies on short eyes have been published (see Table 3). Specifically, Sudhakar et al. (13) retrospectively compared the accuracy of ORA with several formulas in 51 eyes with an axial length of <22.1 mm and concluded that for short eyes it did not differ significantly from the best preoperative biometry-based methods. Our results revealed better outcomes using the Hoffer Q and Holladay 2 formulas, with ORA VLynk being comparable to the SRK/T, Haigis, Barrett Universal II, and Barrett true K formulas (Table 3, *p* > 0.9). Sudhakar et al. (13) also compared the outcomes of the different methods after optimisation in eyes that received a monofocal IOL. They found that although optimisation did change the performance of many of the formulas with regard to the proportion of eyes within ±0.50/1.00 D of the target SE, the differences reported were small and not significant. They indicated that ORA remained one of the best-performing methods but it was not statistically significant to the others. They also discussed the possible factors relating to the poor performance of biometry-based methods for calculating IOL power in short eyes, suggesting that this was related to effective lens position determination, the high powered IOL implanted, and/or manufacturing processes. Soifer et al. (14) also retrospectively analysed 23 highly hyperopic eyes, and Bansal et al. (16) in their prospective study to compare ORA with different IOL power calculation formulas in 65 short eyes (<22 mm) concluded that ORA was more effective in predicting IOL power than Haigis, SRK/T, and Barrett Universal II, although it was equivalent to Hoffer Q. They also indicated that Hoffer Q was superior to all formulas in terms of the percentage of patients within 0.50 D of their target refractions and the percentage of patients going into hyperopic shift. This agrees with the outcomes we found in our series of short eyes (see Tables 2, 3). Analysing the mean absolute error value in detail, our results were similar to those found by these authors: about half a diopter for the SRK/T, Holladay 2, Barrett Universal II, Hoffer Q, Haigis, and ORA VLynk calculation methods (see Table 5).

It has been reported that for eyes with an axial length of <22.0 mm the predictive accuracy is less precise: within ±0.50 D ranged between 21 and 71% (18) and between 45 and 75% (19) as a function of the formula used. In fact, it seems that there is no general consensus on which the best biometry-based formula is for these eyes, since some outcomes indicate that Haigis produced the smallest mean absolute error (19), while others consider Holladay 2 to be more precise (20), others found that Barrett Universal II was the most accurate (21), and yet others that Hill-RBF (22, 23) yielded the lowest numerical error. Our results (Table 2) indicate that all these biometry-based formulas and the ORA VLynk show a mean absolute error ranging from 0.40 to 0.50 D. In relation to the proportion of eyes within ±0.50 D and ± 1.00 D, Table 6 shows that the outcomes of this and previous studies are quite similar when comparing the different methods individually: 40–70% and 80–90% being within ±0.50 D and ± 1.00 D, respectively; we found the highest percentages for the Holladay 2 and Hoffer Q biometry-based formulas.

Raufi et al. (24) retrospectively compared the outcomes of ORA to Barrett Universal II and Hill-RBF 2.0 in a large population (949 eyes) and found that axial length stratification (<22.75 mm, 22.75 to 24.5 mm, 24.5 to 26.25 mm, and > 26.25 mm) did not influence statistical differences in the IOL prediction methods. Thus, if a surgeon were to specifically use Hill-RBF or Barrett Universal II, there would be no advantage gained by supplementing these with ORA. These authors concluded that ORA is, however, still promising in eyes with a history of corneal refractive surgery and in eyes needing toric IOLs, for example. It has also been reported that certain factors, such as speculum-induced pressure, eyelid pressure, and intraoperative corneal changes, may affect the variability of the ORA system (2); additionally, after crystalline lens extraction, variations in the aphakic intraocular pressure, corneal incision, and hydration may also contribute to measurement errors and variable IOL selection (14).

## 5 Conclusion

To our knowledge, this is the first study to prospectively assess the accuracy of the ORA VLynk and preoperative biometry-based formulas in short and long eyes when the same IOL was implanted. The outcomes reported in our study suggest that for long eyes implanted with the same monofocal IOL the ORA VLynk system performs better than all conventional biometry-based formulas. For short eyes, The ORA VLynk appears to perform as well as SRK/T, Haigis, Barrett Universal II, and Barrett true K, although Hoffer Q and Holladay 2 are the most accurate biometry-based formulas. However, the differences between all the calculation methods are small. We believe that this approach reduces undesired postoperative refractive errors and patients with long or short axial lengths could benefit from the use of this technology. Future research should explore the efficacy of ORA VLynk in long and short eyes implanted with premium IOLs, and eyes with corneal diseases, such as keratoconus.

## Data Availability

The raw data supporting the conclusions of this article will be made available by the authors, without undue reservation.
